# Family’ members experiences of their older relative’s alcohol and substance misuse

**DOI:** 10.1080/17482631.2022.2094059

**Published:** 2022-07-03

**Authors:** Aud Johannessen, Kjerstin Tevik, Knut Engedal, Thomas Tjelta, Anne-Sofie Helvik

**Affiliations:** a The Norwegian National Centre for Ageing and Health, Vestfold Hospital Trust, Tønsberg, Norway; b b Faculty of Health and Social Science, University of South-Eastern Norway, Kongsberg, Norway; c Department of Public Health and Nursing, Faculty of Medicine and Health Sciences, Norwegian University of Science and Technology (NTNU), Trondheim, Norway

**Keywords:** Addiction, adult children, alcoholics, caregiver, cohabitant, dependency, family, misuse, next of kind, relatives, spouses

## Abstract

**Background:**

Alcohol consumption in Norway and much of the western world has increased during the past decades, in particular among older adults (> 65 years). Although living with a family member’s alcohol misuse has been shown to have a significant deleterious health impact, research on this topic is both lacking and urgently needed to develop targeted health services.

**Aim:**

To generate knowledge of how family members are affected by their older relatives’ alcohol and other substance misuse problems.

**Method:**

In 2020, 17 individual interviews were carried out with the wives and adult children of older adults with alcohol and other substance misuse problems. Data were analysed using content analysis.

**Findings:**

Analyses revealed two main themes; *the impact of living with psychological stress over time*, and *the impact over time on family relationships and functioning*. Both included four subthemes, representing different dimensions of participants’ experiences of the impact of their older relative’s alcohol and substance misuse.

**Conclusion:**

The challenges family members experienced through ongoing exposure to their relatives’ alcohol and/or other substance misuse increased over time. These experiences had significant negative consequences for their health and life situation.

Alcohol consumption among older adults (> 65 years) in most western countries including Norway has increased during the past several decades (Bratberg et al., 2016; Kelfve et al., 2014; Rao et al., 2015; Tevik, 2019). In Norway, this increase has been highest among those aged 66 to 79 years (Bye & Østhus, 2012), and the gender gap in drinking has decreased (Bratberg et al., 2016; Stelander et al., 2021). Older adults who participated in the North Trøndelag Health study (HUNT), for example, almost doubled their intake of pure alcohol between 1995–1997 (HUNT2) and 2006–2008 (HUNT3; Støver et al., 2012). Pure alcohol consumption among men aged 60–69 years increased from 1.5 litres to almost 2.5 litres per year, while women in the same age group increased their consumption from 0.6 litres to 1.2 litres (Støver et al., 2012). Women also use addictive benzodiazepines and z-hypnotics drugs (psychotropic drugs) more frequently than men (Støver et al., 2012; Tevik et al., 2017, 2019). Some older adults may have sustained their drinking over time, others may have increased their alcohol consumption as a consequence of changes in their social roles and work identities (Emiliussen et al., 2017).

Several countries have implemented alcohol guidelines (Tevik et al., 2021; IARD, 2019). These guidelines do not include targeted advice for older age groups, although most recommend lower levels of consumption for older adults, those with existing health conditions or medications that interact with alcohol. Neither national alcohol guidelines or the Diagnostic and statistical manual of mental disorders (DSM-5) provide a definition of elevated consumption levels for older adults (Hoffmann & Kopak, 2015). Nevertheless, there is consensus that older adult should limit their alcohol consumption to one unit per day or less, and that even very low levels of alcohol consumption may be hazardous for frail older adults (Fillmore et al., 2007; Johannessen et al., 2015, 2021). This study focusses on participants’ experiences of their relative’s alcohol and/or substance misuse problems.

It is estimated that 100 million individuals misuse alcohol or other substances worldwide, although results are not reported by age (Orford et al., 2013; Rao et al., 2015). Rao et al. (2015) found that alcohol and substance misuse among older adults is associated with a significant negative health impact on their families. A recent scoping review suggests that for decades, these family members have been implicated in the pathology of alcohol misuse, rather than recognized as a high risk population in need of treatment and support (Di Sarno et al., 2021). However, this review did not report variation by age, gender or relationship type (Di Sarno et al., 2021). Family members living apart from a relative with alcohol misuse problems may also experience negative health outcomes (Newton et al., 2016). Studies have shown that children living with alcohol abuse in the family experience a range of negative outcomes (Nordlie, 2003), including post-traumatic stress disorder (Alisic et al., 2014), interrupted sleep patterns and physical and mental exhaustion (Kristiansen & Myhra, 2012, p. 51).

A Norwegian report on family members referred to specialist treatment services due to a relative’s substance misuse problems found that most were women (Kristiansen & Myhra, 2012, p. 51). Typically, these women had lived with their partner’s alcohol or substance misuse for several years, and approximately two thirds reported that they first asked for help after living with alcohol misuse over time and following a specific critical incident (Kristiansen & Myhra, 2012, p. 51). Lindeman et al. (2021) suggest the delay in seeking help may result from the family members’ experiences of shame, guilt, exhaustion, grief, and perceived lack of support (Birkeland, 2019; Lindeman et al., 2021; McCann & Lubman, 2018b; O’Shay-Wallace, 2019).

Few studies have examined how family members experience their older relative’s alcohol and substance misuse. This knowledge is urgently needed to develop targeted health services and interventions (Duckert et al., 2008; Johannessen et al., 2015, 2021; Kjosavik, 2012).

## Aim

To generate knowledge of how family members are affected by their older relatives’ alcohol and other substance misuse problems.

## Methods

### Design

Qualitative individual interviews were carried out with close family members of older Norwegians with alcohol misuse problems, some of whom also misused psychotropic drugs (Berg & Lune, 2012).

### Participants

Eighteen participants were purposive recruited via telephone by health personnel and through voluntary organizations. Two hospitals in the field of alcohol and substance misuse disorders recruited six patients’ next of kin. Next of kin were also recruited from three voluntary organizations working with families of individuals with alcohol and/or substance misuse disorders in mid and southern Norway. Eleven participants were recruited from these organizations. One participant dropped out, and a total of 16 women and one man participated in interviews. Eleven were daughters, one was a son, four were wives and one cohabited with an older adult with substance misuse problems. The cohabitant is referred to as a wife in the text hereafter. The adult children were aged between 31 and 57 years, and the wives between 53 and 66. The partners or parents were aged between 62 and 80 years at the time the interviews were undertaken. All participants had lived with the relative for an extended period of time. Further participant characteristics are listed in Table I.

**Table I.** Overview of the characteristics of the wife’s, the adult children and the older persons with a misuse of alcohol and/or drugs.

Wife’s and adult children					The older persons with a misuse		
Gender^1^ (age)	Relation (Name)^2^	Age when the misuse started	Civil status	Own children	Relations (Age)	Civil status	Type of misuse
W (47)	Daughter (Linda)	6 years	Married	2	Mother (77)	Divorced	Alcohol and drugs^4^
W (32)	Daughter (Sofia)	4–5 years	Single	0	Father/Mother (74/73)	Divorced	Alcohol and drugs^4,5^
W (53)	Wife (Louisa)	Always	Married	2	Husband (62)	Married	Alcohol and drugs^4^
W (59)	Wife (Rachel)	Always, but escalated past 25 years	Married	1	Husband (62)	Mariried	Alcohol
W (49)	Daughter (Carol)	8 years	Single	0	Father (85)	Married with the mother	Alcohol
W (36)	Daughter (Ann)	5–6 years	Married	2	Father (70)	Married with the mother	Alcohol
W (57)	Daughter (Mary)	Early childhood	Married	1	Father/Mother^3^ (Death/80)	Divorced	Alcohol
W (31)	Daughter (Nancy)	Teenager	Single	1	Mother (63)	Married with the father	Alcohol and drugs^4^
W (40)	Daughter (Catherin)	6–7 years	Divorced	2	Mother (65)	Cohabitant (Divorced from the father)	Alcohol and drugs^4^
W (43)	Daughter (Laura)	6–7 years	Single	0	Father/Mother (65/66)	Divorced	Alcohol
W (31)	Daughter (Michel)	12 years	Single	1	Father (67)	Divorced	Alcohol
W (50)	Daughter (Ruth)	6–7 years	Divorced	2	Father/Mother (Death/72)	Divorced	Alcohol
W (35)	Daughter (Camilla)	Early childhood	Cohabitant	0	Father/Mother (64/death)	Divorced	Alcohol and drugs^4,5^
W (66)	Wife (Cecilie)	Always, but escalated the past 15 years	Married	1	Husband (68)	Married	Alcohol
W (65)	Cohabitant (Grace)	Always	30 years	2	Cohabitant (68)	Married	Alcohol and drugs^4^
W (57)	Wife (Olivia)	9 years	15 years	2	Husband (67)	Married	Alcohol
M (47)	Son (Thomas)	Early childhood	Married	2	Father (75)	Married	Alcohol

^1^Woman = W; Man = M; ^2^The participants pseudonyms; ^3^First the father and later also the mother; ^4^Drugs that were used was prescribed addictive psychotropic drugs; ^5^Only the mother was using drugs.

### Interviews

Ten interviews were conducted in Norwegian by AJ via telephone and five through Facetime or Skype due to the Covid 19 pandemic. Two interviews were carried out face-to-face, one in a private home and one in a meeting room at a voluntary organization.

### Data collection

An interview guide based on three thematic questions was applied: 1). How do you experience your family member’s alcohol and/or psychotropic drug use? 2). How has this affected/does that effect you in everyday life? 3). How does it affect your family?

Follow-up questions were formulated depending on participants’ replies. New questions were recorded in field notes and used in subsequent interviews to enrich, elaborate and expand on the interview data (Berg & Lune, 2012; Lincoln & Guba, 1990).

A professional typist transcribed the Norwegian recorded interviews verbatim within two weeks of each interview. Quality control checks were performed by AJ listening to the interviews while reading through the transcripts. Interviews lasted between 13 and 38 minutes (in total 450 minutes and mean 24 minutes).

### Analysis

Data were analysed using manifest qualitative content analysis (Graneheim & Lundman, 2004). Transcripts were read carefully several times to establish an overall impression. No substantial differences as regard to content or depth were identified. Next “meaning units”, words and sentences expressing a central meaning were identified and systematically condensed without abstracting from the original text. At the second stage, the condensed units were labelled with a code stating their content. In the third and final stage, analytical themes and subthemes were created. These consisted of groups of codes reflecting the central themes of the interviews. Special attention was paid to establishing clear differences between and similarities within codes and themes (Graneheim & Lundman, 2004). Authors AJ and A-SH had principal responsibility for the analysis, but the process was discussed between the authors.

### Ethics

The study followed the ethical principles outlined in the Helsinki Declaration (World Medical Association, 2013) and was presented to the Data Protection Service which determined the Norwegian Medical and Health Research Act did not apply. Thus, it did not need an approval from the Regional Committees for Medical and Health Research Ethics. Informed consent was collected from participants after they had received oral and written information and before the interviews took place.

## Findings

Two main themes emerged: “The impact of living with psychological stress over time”, and: “The impact on family relationships and functioning”. Both included four subthemes, representing different dimensions of participants’ experiences of their older relative’s alcohol and substance misuse, see, Table II.

**Table II.** The main themes and subthemes that emerged from the structural analysis.

Main themes	Subthemes
The impact of living with psychological stress over time	*Chronic feeling of insecurity and fears for personal safety*
	*Coping with the particular challenges of alcohol misuse in old age*
The impact over time on family relationships and functioning	*Continuing and escalating challenges with family relationships*
	*Continuing extrafamilial and social challenges*

### The impact of living with psychological stress over time

#### Chronic feeling of insecurity and fears for personal safety

Participants frequently felt insecure in their everyday lives, and the resulting stress eroded their trust both in their parents or partners and in others. They described feeling “shut off” from their emotions, resulting in problems at school during childhood and/or sick leave from work in adulthood. Some also struggled with their own drug and/or alcohol use. Over time, these negative feelings affected their mental health and functioning. One wife stated: *I am an active, open-minded, social, and healthy person, but during the past few years I have been experiencing poorer mental health. I sleep badly! Last year I had an anxiety attack. I have no doubt that it is related to the fact that I have worried about his health for so long*. Interview with Louisa.

A daughter said: *I’ve been a revolving door psychiatric patient for years. I’m left with nothing in a way … I have no education, I have had to call in sick, things have got in the way and so … There are some long-term consequences, poverty and lack of education and work because of all those years. But all in all, I think I’m doing very well now*. Interview with Michel.

The adult children also recalled adults in the neighbourhood who had looked after them in childhood but had not reported the family’s situation to health services or schools. These adults were valued sources of emotional support, and many of these relationships had been sustained over time. One daughter stated: *These people have been there for me and been a rock in my life, like my parents should have been. Everyone who has experienced these kinds of painful things deserves someone who stands up for them and cares*. Interview with Ann.

Participants reported that their bodies reacted variously after years living with the affected family member: exhaustion, fatigue, self-harm, eating disorders and post-traumatic stress disorders were mentioned. Much of their anxiety resulted from uncertainty regarding when the next period of alcohol abuse would start. Participants had been subjected to violence and aggressive behaviour, including shouting, throwing objects, slamming doors, harassment, threats, and insults. Incidents of sexual abuse were also described. One daughter said: *I’ve shut down emotionally, flipped straight into anger and physical aggression, and have experienced significant consequences in adulthood. I’ve had a lot, a lot of anxiety. It’s not always bad, but there have certainly been some very dark days. I’ve been in therapy for a very long time, but I got help*. Interview with Ann.

Participants held a lot of anger, and described feeling guilty. The chaotic nature of their family lives resulted in difficulties building trusting relationships with others both in childhood and more recently. Most had yet to come to terms with the long-term impact of alcohol and/or substance use in the family: holidays, birthdays and family occasions remained a cause for anxiety and uncertainty rather than celebration.

The unpredictability of the affected family member’s behaviour also caused participants anxiety. One daughter said: *It was very difficult. I couldn’t bring friends home because of the different things my mother said and did. So, I had to avoid bringing friends home. Consequently, later in life, it hasn’t been easy for me to trust people. I think about this very often*. Interview with Mary.

Participants attempted to hide their parents’ drinking from others in childhood due to the stigma associated with alcohol misuse problems. Several avoided bringing friends home from school, and many recalled feelings like an outsider. One daughter said: *Two of my close friends were very surprised when I told them how things really were at home, because I’d hidden it well. Then I got anxiety and … I think I first got the reactions then. Then I got psychological help, to a certain extent. I think the first time I got psychological help was when I was 22, maybe. Pretty late*. Interview with Sofia.

The wives also foregrounded the stigma associated with alcohol misuse and said this made it difficult to seek support from colleagues, friends, and professionals. They were concerned about not being understood or well received. However, some had a trusted friend they could talk with openly. One wife stated: *I have some friends that know about my husband’s drinking, and that has made it easier*. Interview with Cecilie.

All participants highlighted the importance of understanding and professional support from someone they could trust. Some received help from general practitioners, psychiatrists, psychologists, or addiction clinics. One daughter said: *The way he [General Practitioner] treated me, took me seriously, listened to what I said, and has, consistently, gives me security and a feeling that this is not just something I’ve invented. Yes, it’s been very important*. Interview with Ruth.

#### Coping with the particular challenges of alcohol misuse in old age

Participants described increasing concerns that an accident may result from intoxication as the affected family member aged, and wives expressed concerns their partner would leave, the drinking would increase, or injuries be sustained and their health diminish further. One wife stated: *When he’s in that mood he threatens me with divorce. It’s a paradox. Like he screams: “wolf, wolf” because he has threatened to divorce me a hundred times, but never done anything about it*. Interview with Carol.

Another wife experienced similar anxieties: *What has been most difficult for me has been a very strong unrest, and fear. When I’m not at home, I’ve been afraid of what’ll happen. I’m afraid what’s going to happen when I’m not there—that he’s going to hurt himself or something. I’m afraid he’ll forget to turn off the hob, so the house burns down, things like that*. Interview with Linda.

All participants described feeling responsible for the affected family member’s personal safety as they aged. A son stated: *I had a lot of responsibility when I was a child. I had to go outside and look for him, find him, help him home. I think that has affected me. I feel like I always must look after him*. Interview with Thomas.

Despite this, the adult children would attempt to maintain a physical and mental distance between themselves and their parent, while still feeling responsible for them. They were concerned both for their health and physical safety. A daughter mentioned the following concerns for her mother: *I’m afraid she’ll fall when she calls me and I can hear she’s intoxicated. I can’t help her because I don’t live there. I’m glad I have an aunt that can go and look after her sometimes*. Interview with Sofia.

Caring for parents and partners became increasingly difficult as they aged, regressing to what participants described as a “childlike” state, becoming selfish and absolving themselves of responsibility for their behaviour. It also became increasingly difficult to see them as patients in need of care. One daughter describes her caring role as follows: *I feel that I must look after her as she gets older and needs more help. Practical help, but also help with services. It’s more to take on now than it was before*. Interview with Linda.

Participants also experienced feeling powerless, that there was nothing they could do to ameliorate the negative consequences of their family member’s alcohol or substance use. Some attempted to regulate their parent or partner’s drinking. However, this put additional strain on family relationships, and rarely worked. Even when the affected family member had agreed to limit their drinking, the agreement was seldom adhered to. One daughter said: *I buy beer for my mother now so that she doesn’t drink spirits*. Interview with Mary.

A wife regulated access to sleeping pills rather than alcohol, and said: *The times he drinks and has managed to get hold of sleeping pills without me giving them to him, I know from experience this combination is potentially life-threatening, and I’m afraid that something bad will happen while I’m not around*. Interview with Louisa.

Attempts to regulate the affected family member’s hazardous substance use became exhausting. Some participants took steps to mitigate their feelings of powerlessness by refusing to take responsibility for their relative’s health. One daughter said: *I’ve always been good with visiting my mother, but the past few years I’ve made a choice to live my life. It makes me feel calmer*. Interview with Laura. However, few were ultimately able to avoid feeling responsible for their parents.

### The impact over time on family relationships and functioning

#### Continuing and escalating challenges within the family

The wives and adult children experienced difficulty establishing and maintaining emotional distance from their husband or parents, and these challenges increased as they aged, their need for care increased and their health declined. The wives stated that their partner’s alcohol or substance misuse created conflicts in the family. The adult children also described how their roles in the family changed as they effectively assumed the role of parents, becoming responsible for their own mother or father. One daughter said: *I used to describe moving away from home as being like leaving prison, but I was concerned for my siblings, and they very quickly let me know they felt I had forgotten them when I went to college while they were left behind and had a hard time. So, I went back home every weekend to check up on them. There was no one at school that took the time to understand or see what we went through. I’ve found that’s still the case. Teachers don’t care*. Interview with Carol.

Participants also described how their relative impacted on other family relationships. The adult children described how members of the extended family were pitted against each other, with siblings and other family members assuming different perspectives on the situation, which generated conflict. One daughter said: *It’s been very difficult to keep her [mother]out of my life. I’ve tried … twice, but then she contacts other family members and somehow becomes part of my life again. None of the family have managed to maintain those kinds of boundaries. But now everyone thinks I’m the big bad wolf. I’m letting go of the drama. It’s painful to lose the whole family because of my mother’s drinking, but for me this decision has been important, and it’s the right choice*. Interview with Catherin.

The adult children also tried to regulate contact with their parents to ensure the rest of the family could enjoy special occasions without their relative being present. However, this was not always possible. One wife stated: *Yes, it’s been the classic: Christmas, Easter, holidays. And what is particularly painful is that it’s happened when we’ve been celebrating our daughter’s birthday. He was drunk before the party started. He’s still like that now. It’s hard for our daughter to come home because she doesn’t know how things are at home and when it’s all going to kick off*. Interview with Rachel.

Participants described how they protected themselves and their children from their grandparent’s addiction. Contact was regulated in different ways: by avoiding telephone calls or meeting in circumstances where the relative was likely to be intoxicated. One daughter said: *Yes, she’s their grandmother, but she will never look after my kids. She’s met them, but only when I’m there*. Interview with Nancy.

#### Continuing extrafamilial and social challenges

Participants also regulated access to alcohol in social contexts. Only close friends who knew about the ongoing alcohol misuse problems would be invited to the family home, and those unwilling or unable to abstain from drinking were excluded. One wife states: *At last, there’s a certain openness on this topic, because I’ve been telling his friends this is a problem for almost for 20 years, or at least 15. They just thought I was exaggerating, made me the big bad wolf. I get a bit more understanding now*. Interview with Rachel.

Participants also experienced limited opportunities to discuss their relative’s alcohol misuse with others. The subject had been tabooed at school and was taboo at work, and participants lacked confidence that teachers, co-workers, neighbours, family members or other adults that knew about their parent or partner’s alcohol misuse would modify their behaviour accordingly, even when they had witnessed the parent or partners’ drinking. Participants were therefore reluctant to disclose or discuss their experiences with others, and rarely received help from support services, schools, or through their social networks. One daughter said: *I think health centres for children and specialist community public health nurses should talk more openly about alcohol misuse problem. Teachers and other nursery staff too. I think they’re too careful about these things. It’s tragic that alcohol abuse is still so difficult to talk about, because it’s so important to get help early*. Interview with Ann.

Some participants received support from addiction clinics while their relative was undergoing treatment. Others had contacted addiction clinics for help. Most experienced the support they received as helpful, as addiction was considered a difficult topic to raise in more informal settings. Group therapy was popular, providing participants with an opportunity to share their stories with others in a similar situation. But participants emphasized professional help should have been made available earlier. One daughter stated: *The stories that are told by others of addiction in the family, it gives you a feeling of not being mad, or having invented things … That’s probably what has been and remains most helpful, to talk about your own experiences of being the child of a problem drinker helps*. Interview number Catherin.

Voluntary organizations were also identified as an important source of support, and opportunity to meet with others in a similar situation. Accessing support was an ongoing process. Participants struggled in isolation, but found sharing their experiences helpful, and a way to challenge taboos. One daughter said: *If I had to choose what was most important it would be the user organization I’m a part of now. It gives me an opportunity to see and meet other people that have been through similar experiences. People who are vulnerable in the same way*. Interview with Ann.

The opportunity to help others was also identified as meaningful. One daughter stated: *I’ve volunteered in the organization for about 10 years. That’s been important to me. Just to help others, but it’s not something you can do all the time, because then these experiences take up too much space in your life*. Interview with Linda.

## Discussion

Findings are discussed in order of themes (Table I) to contribute to a broader understanding of how family members are impacted by their parent or partner’s alcohol misuse problems.

Psychological stress over time resulted in persistent feelings of insecurity and fears for personal safety. This resulted in difficulties building trusting relationships with others. Exhaustion, fatigue, self-harm, eating disorders, and posttraumatic stress disorders were identified as consequences of alcohol misuse in the family, consistent with findings from other studies (Alisic et al., 2014; Kristiansen & Myhra, 2012, pp. 52–53; Nordlie, 2003; Orford et al., 2013). Participants also reported feeling disconnected emotionally: problems at school or sick leave from work were reported, and alcohol misuse problems in adulthood, which has also been reported previously (Almquist, Bishop, Gustafsson & Berg, 2020; Marino et al., 2018; Rospenda et al., 2010).

Participants reported anxieties associated with uncertainty when the next episode of alcohol misuse would start, and had experienced violence, aggression and sexual abuse. These experiences are commonly reported in the literature, and appear not to decrease with age. In spite of these experiences, help was not considered to be readily available or easily accessed (Hellum et al., 2021; McCann & Lubman, 2018a). The associated psychological stress, insecurity, and anxiety have clear mental and physical health implication over time, as documented in several studies and a recent review (Di Sarno et al., 2021; Kristiansen & Myhra, 2012, pp. 52–53; Nordlie, 2003; Orford et al., 2013). This underscores the importance of moving beyond notions of enabling and co-dependency to acknowledge family members of problem drinkers as a high-risk population in need of treatment and support (Di Sarno et al., 2021). Social influences seem to play an important role in help-seeking behaviour (Eubanks Fleming, 2016). The family members in the present study also found ways to cope, albeit often by keeping their relative at a distance.

Our study found participants hid their relative’ alcohol misuse from others. The subject was considered taboo. This stigma made it difficult to seek support from colleagues, friends, or to seek professional help, as reported elsewhere in the literature (O’Shay-Wallace, 2019). O’Shay found that stigma may be managed through avoiding, denying, and ignoring problems, which was not reported in the present study. However, participants highlighted the need for professional support and experienced access to support as lacking or severely limited, including as their relative aged. It is well-documented that family members of relatives that misuse alcohol or drugs need professional support. Action should be taken to develop support programmes at various levels of the health care system, and to challenge taboos and associated stigma. The family’s needs should be made visible and not remain invisible (Lindeman et al., 2021; McCann & Lubman, 2018b; McCann et al., 2019).

Participants also had concerns that accidents may occur while their older relative was intoxicated. Another study found that intoxication increased the risk of accidents among older adults, and highlights a need for effective preventive strategies to reduce heavy episodic drinking (Bye et al., 2021). Regulating access was one strategy highlighted in this study: however, this was not always successful and created conflicts in the family. We believe such strategies are insufficient, and that these families need professional support. Family members cannot be accountable for the health, safety and wellbeing of their ageing relatives. The challenges associated with this caring role can also cause significant difficulties in adulthood (Tedgård et al., 2019).

The roles in the family changed over time. In adulthood, some children effectively assumed a parental role in looking after their siblings and parents. These role transitions need to be identified sooner, and measures put in place to mitigate the long-term impacts (Tedgård et al., 2019). Our study also showed how alcohol misuse problems affected family relationships, which could impact on future generations. Further, participants protected themselves and their children by avoiding visits or contact in situations in which their relative was likely to be intoxicated. A study points out that all family members suffer when one member has an alcohol misuse problem. It is important to recognize that the family as a whole need help as early as possible, both to mitigate the effects of harmful alcohol use and prevent the intergenerational transmission of alcohol related harm (Ólafsdóttir, 2020).

Participants experienced difficulties sustaining relationships with others as only close friends knew about the ongoing alcohol misuse and could be invited into their home. Talking to others about the misuse was considered taboo because those that knew about their parents’ or partners alcohol misuse problem did not modify their behaviour, even when they had witnessed their drinking. The associated stigma contributed to the anxiety and psychological stress experienced by family members. Newton et al. (2016) found that many people seeking support for depression and anxiety were related to an individual with alcohol or other substance misuse problems. These people also need to be visible and be supported (Lindeman et al., 2021; McCann & Lubman, 2018b; McCann et al., 2019). The support should be provided early in life and before the relative is enrolled for treatment in specialist health care. Group therapy and peer support were considered beneficial, and these measures should also be implemented at primary care level.

Voluntary organizations were also experienced to be helpful. The possibility to meet others in a similar situation at these organizations or clinics had been and remained particularly valuable for participants. We also found that help seeking was represented as a learning curve, or an ongoing process, and key to coming to terms with the impact of problem drinking in the family on their daily life and functioning. Helping others was also experienced as meaningful, as reported elsewhere in the literature (Buber, 1923; Johannessen, 2012).

### Strength and limitations

We believe purposive sampling helped validate the findings, although difficulties recruiting men limited the diversity of the final sample. Almost all participants were women, primarily daughters (Patton, 2002).

A strength was that to enhance the trustworthiness of the data, quotations were presented in the text, representing 14 of the 17 participants. In addition, data were analysed and discussed among the authors (Lincoln & Guba, 1990). Although our findings cannot be generalized, we argue that they can be valuable to family members, health personnel, and volunteers that work with family members of older adults with alcohol or other substance misuse problems.

The COVID-19 pandemic precluded face-to-face interviews. Fifteen interviews were therefore carried out by telephone, Facetime and Skype. One of these interviews only lasted for 13 minutes. We nevertheless considered the information from all interviews (lasting for a total of 450 minutes mean 24 minutes) to be valuable. Another potential limitation may be presented by the relatively unstructured topic guides, which included only three main questions. We believe, however, that recording follow-up questions in field notes and deploying these in subsequent interviews enriched and nuanced the interview data (Berg & Lune, 2012; Lincoln & Guba, 1990). The interviews and transcripts were also generated in Norwegian and translated into English. In this process a weakness could appear, but we believe that have managed to do this in a truthful. We have been careful to preserve all quotes in their original contexts.

## Conclusion

The challenges and psychological stress on family members of relatives misusing alcohol or other drugs persist over time, and may increase as the relative ages and suffers from other health related issues related to the ageing process. Relatives try both to support and to distance themselves from their families. They need to be made more visible and offered support and help from early in life. Appropriate support programs should be organized at different levels of the health care system.
